# Rétrograde venous perfusion (RVP) for intraçtable venous leg ulcers: a retrospective analysis

**DOI:** 10.1186/s40001-025-02651-y

**Published:** 2025-05-17

**Authors:** Alaa Sharabi, Mohammed Abd-Eltawab, Sherif A. Sharabi, Mai A. Elkalla, Yossef N. Abdelbaky, Mohamed A. Abou Yossef, Ahmed Mousa

**Affiliations:** 1 Department of Vascular Surgery and Endovascular Therapy, Al-Hussein University Hospital, Faculty of Medicine for Males, Al-Azhar University, Darrasa, Cairo, 11633 Egypt; 2 Department of Vascular Surgery and Endovascular Therapy, Faculty of Medicine, Assiut University Hospital, Al-Azhar University, Assiut Branch, Assiut, Egypt; 3 Faculty of Medicine, Bader University Hospital, Helwan University, Helwan District, Cairo, Egypt

**Keywords:** Chronic venous leg ulcers, Intractable venous leg ulcers, Long-standing venous leg ulcers, Resistant venous leg ulcers, Nonhealing venous leg ulcers, Retrograde venous perfusion (RVP) procedure, Healing of chronic venous leg ulcers

## Abstract

**Background:**

Retrograde venous perfusion (RVP) is a minimally invasive procedure in which the limb circulation is isolated by the application of a proximal limb tourniquet, followed by the administration of specific medications through a distal limb vein. This allows these drugs to pass in the reverse direction to reach the ulcerated area of the affected limb. The aim of this study was to evaluate the safety, feasibility, and efficacy of RVP, for the management of long-standing intractable chronic venous leg ulcers (CVLUs).

**Methods:**

A 4-year retrospective study took place from January 2021 to January 2025. All patients who underwent the RVP technique were included in the study. These patients had chronic, intractable, long-standing, nonhealing, venous leg ulcers. They were classified into two groups. Group I included those who underwent RVP (treated group). However, group II was treated with standard compression therapy (control group). A paired-samples *t* test was performed to compare the studied groups. Kaplan–Meier survival analysis was performed for patients who were free from ulcer recurrence or nonhealing after the RVP technique.

**Results:**

During the 4-year study period, 384 patients were retrospectively analyzed. A total of 75% (*n* = 288) of the participants were females, and 25% were males (*n* = 96). The median age was 37.26 ± 4.2 years. Ulcers ranged between 30cm^2^ and near circumferential. The median ulcer duration was 18 ± 14.4 months. The mean number of RVP sessions was 26.78 ± 7.6, whereas the mean session time was 213 ± 49 min. A reduction in ulcer size/complete healing was achieved in 96.9% of the patients in group I vs. 68.8% of those in group II.

**Conclusions:**

Compared with the standard compression technique, RVP therapy may be considered an effective and feasible technique for treating intractable venous leg ulcers. It offers shorter periods of therapy with a high success rate in reducing ulcer size/complete wound healing within a short period of time. This therapeutic option may provide essential evidence to reduce the negative social and economic impact on affected populations.

## Background

Chronic venous leg ulcers (CVLUs) may be defined as the loss of continuity of the surface epithelium on the distal part of the leg because of venous hypertension [1]. CVLUs are usually located on the distal third of the leg (gaiter area), especially on or immediately above the medial malleolus. However, it may also appear posteriorly or laterally. If it becomes large enough, it may involve most/whole leg circumference [2]. Moreover, poor/resistant healing of CVLUs is commonly attributed to either local or systemic factors. Local factors include the presence of necrotic tissue, infection, tissue hypoxia, repeated trauma, and poor hygiene. While systemic factors may include diabetes and obesity with increased body mass index [3]. The incidence of CVLUs ranges between 0.06 and 2%. Furthermore, the prevalence of more severe ulcers may be classified as C5, C6, and C6r stratifications according to the Clinical, Etiological, Anatomical, and Pathological (CEAP) classification, which may reach up to 1.5% [4]. Intractable venous leg ulcers are usually associated with extensive disability, socioeconomic impact, and serious psychosocial morbidity that significantly reduce patient’s life quality [5]. Furthermore, compression bandage therapy is considered the main form of venous ulcer care, both for the highest rate of promoting ulcer healing and lowering the incidence of its recurrence. Moreover, compression therapy minimizes venous hypertension, by reducing lower leg edema and consequently increasing venous return [6]. However, compression therapy may be aided by interventional venous procedures to eliminate superficial venous reflux [7]. Venous interventional procedures are recommended for truncal vein reflux, superficial varicosities affecting the venous network on the peri-ulcerative area, and to manage perforator incompetent in selected cases. These technical procedures, if performed promptly at an early stage of the disease, will have an obvious reduction in the time of wound healing [8]. In the current study, we reported an innovative, novel, and feasible technique, namely, retrograde venous perfusion (RVP). It comprises the intravenous injection/infusion of pharmacotherapeutic agents. It may be used as an adjunct to local wound dressing and limb compression bandage procedure. This innovative technique in combination with local limb compression may be adopted for the management of chronic non-healing resistant VLUCs with excellent results. At rest, RVP, when applied regionally, may provide sufficient oxygenation to the peripheral tissues through venocapillary networks. In these situations, it may be an effective, feasible, simple, and useful measure for decreasing the ulcer size and consequently promoting complete ulcer healing and avoiding the definitive complications that may threaten the limb. In addition to improving patients’ life quality, the venous clinical severity score (VCSS), as well as the health-related quality-of-life score (HRQLS).

## Methods

After the approval of our institute’s research board and ethical committee, a 4-year retrospective analysis took place between January 2021 and January 2025. Patients were classified into two equal and comparable groups. GI 50% (*n* = 192) included patients who received the RVP therapy preceded by local ulcer dressing using a combination of MEBO ointment and ORACURE gel. In addition to the application of a three-layer limb compression bandage (treated group). However, the remaining 50% (*n* = 192) stratified into GII underwent local ulcer dressing using the previous combination, followed by application of the standard four-layer compression bandage system (control group). All patients received wound care 3 days/week. This wound care consists of ulcer cleaning with 0.9% normal saline, a regular wound toilet, surgical debridement of necrotic tissue in the floor/edge of the ulcer if needed, and topical wound therapy. The study was adopted to treat patients with C5, C6, and C6r according to the CEAP stratifications and their updated guidelines [9]. Also included in the study, patients with recurrent ulcers after they have healed following optimal therapy within a 12-month period. Patients with chronic leg ulcers who had previously been treated with failed/rejected skin grafts. Patients with post-thrombotic syndrome. Patients with circumferential leg ulcers, as well as those patients with resistant venous leg ulcers to heal within 2–3 months of standard compression therapy were also included in the study. All patients underwent full history taking, and a clinical vascular examination of the lower extremities that revealed the manifestations of chronic venous insufficiency, including edema, hyperigmentation, lipodermatosclerosis, and lower leg ulceration. Patient demographics and risk factors were thoroughly retrieved including age, sex, and body mass index (BMI). All patients were evaluated for concomitant peripheral arterial disease (PAD). However, those with absent distal pulses and having an ankle/brachial index (ABI) between 0.7 and 0.5 were excluded from the study. Patients with leg ulcers other than venous, pregnant females, patients with rheumatoid arthritis, patients with connective tissue disorders or vasculitis, patients with blood diseases, patients with malignancies, patients on medications that might impair ulcer/wound healing, and patients with active superficial thrombophlebitis were also excluded from the study.

### Different methods of ulcer/wound measurement

Different methods for wound measurement are available. The most widely used formula for an ellipse is: area = length (L) × width (W) × 0.7854, which is called the Kundin method formula [10]. However, the rural-based method of wound measurement is a more reliable and useful way to measure circumferential lower leg ulcers or oval-shaped ulcers. In the present study we selected the Kundin method formula for the measurement of small and easily measurable ulcers. While the rural-based method was adopted for circumferential lower leg ulcers [11].

### Indicators of progressive ulcer healing

As a predictor of progressive ulcer healing is 20–30% reduction in the percentage surface area following the first 3–4 weeks of ulcer management, it is often considered as a good indicator of a proper response to ulcer healing. However, rapid wound healing requires a healing speed of ≥ 1 cm^2^/week [12].

### Technical procedure for retrograde venous perfusion (RVP)

**Fig. 1:**
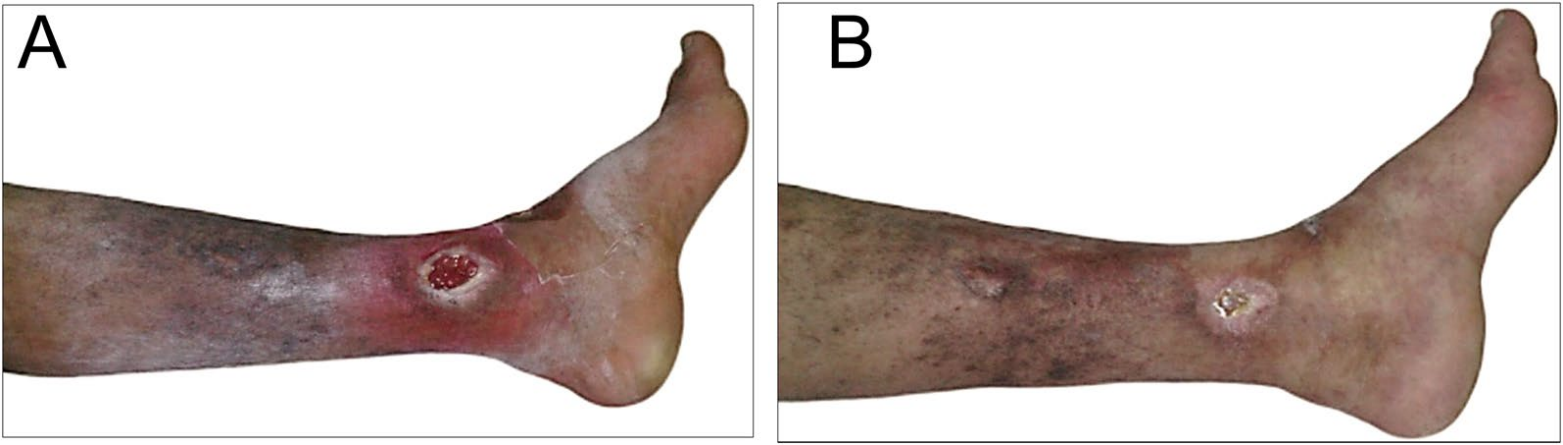
Venous ulcer before (**A**); and after 45 days of RVP sessions with the formation of healthy granulation tissue, creeping epithelium and near wound closure (**B**)

Before the RVP was started, pre-procedural cannulation was performed. A proper-sized intravenous cannula was inserted into any visible vein in the dorsum of the foot (Fig. 1). The cannula size ranged between 18 and 22 gauges.

**Fig. 2:**
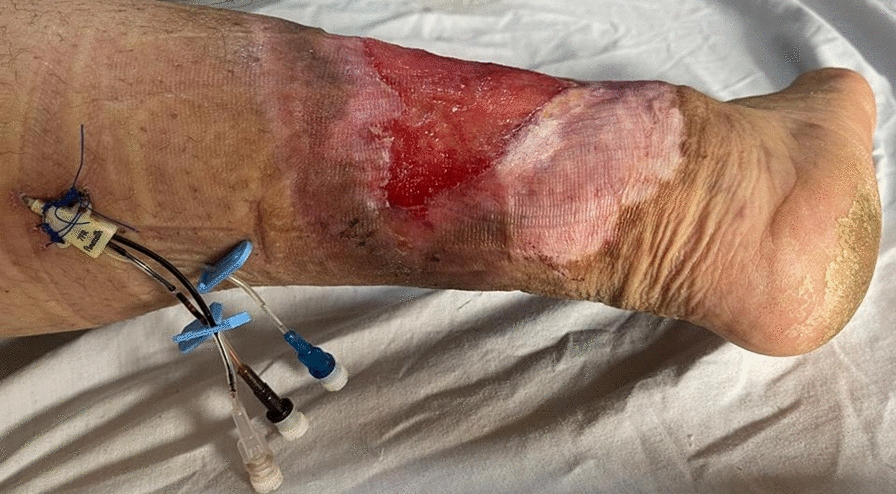
Central venous cannula inserted into the short saphenous vein in patient with circumferential leg ulcer

However, in selected cases with weak and small caliber/absent dorsal foot veins, a central venous cannula (CVC) was inserted into the GSV either surgically with a venous cut down or under ultrasound guidance below the knee and proximal to the ulcerated area (Fig. 2). The insertion of the CVC in the greater or lesser saphenous vein may provide constant and long-term venous access for the completion of RVP sessions.

### Ulcer cleaning and topical wound therapy

The ulcer was cleaned, and topical therapy implies the application of a combination of 0.25% MEBO cream (Gulf Pharmaceutical Industries, Ras Al Khaimah, UAE), and MEBUCAIN gel (Socratec, Erfurt, Germany).

### Three-layer compression system

However, in the current study, we applied a three-layer compression bandage before RVP injection/infusion therapy to prevent postprocedural edema of the treated limb. All compression bandages were applied according to the manufacturers’ recommendations and instructions. The compression bandages were changed before every RVP session (i.e., 2–3 days after the last session), or where there was excessive discharge soaking the bandage dressing, and if needed, wound debridement and systemic antibiotic therapy were prescribed as indicated by culture and sensitivity tests [13]. However, the modified Unna boot [14], made with moist 60% glycerin paste, and 40% zinc oxide was spread over a gauze pad, which may be applied directly over the wound. This mixture was kept in place for 5–7 days, then removed and reapplied if needed. The modified Unna boot was applied in selected patients with cellulitis and superficial thrombophlebitis.

### Four layers vs. different layers of compression bandage systems

In the RVP treated group we used a three-layer rather than a four-layer compression bandage that was applied to the control group. This is based on the literature that healing of venous leg ulceration using a three-layer compression system is more effective than the four-layer compression technique [15, 16]. However, other literature reports that the healing of VLUCs using a two-layer bandage system has a similar effect as the four-layer compression system and is more cost effective [17]. Others revealed that the choice of any bandages technique (either four-layer or short stretch bandages) does not affect pain, recurrence rates, healing times, or HRQLS. From a practice perspective, this good news for patients’ care may allow individuals and practitioners to choose the best compression technologies based on clinical situations and circumstances [18]. On the other hand, a prospective study performed by Scriven et al. [19] showed no differences between the regular Four-layer compression and the short stretch bandages. They reported proper and efficient venous ulcer healing in both types of compression regardless of ulcer duration, size and the pattern of vein reflux. For the previous evidence, using different layers of compression bandages may be used in both groups. It may not significantly affect the results that were obtained.

### Name of the bandages and their types

The four-layer compression bandage system was initially developed at the Charing Cross Hospital in the UK. While the commercially available and widely used four-layer product in Canada is Profore® (Smith & Nephew Medical Ltd.) [18]. It consists of (1) an orthopedic initial padding wool layer (Velband; Johnson & Johnson, Arlington, Tex; or Sofban; Smith & Nephew, Solothurn, Switzerland), (2) a second short stretch crepe bandage layer; (3) a third long stretch bandage layer (Elset, Seton, England), and (4) a fourth cohesive middle stretch bandage layer (Coban; 3M, Rüschlikon, Switzerland) [20]. Randomized controlled clinical trials comparing the original Charing Cross System with Profore™ (Smith & Nephew) revealed no significant differences within 24 weeks. However, a small benefit toward Profore™ was reported at 12 weeks [20, 21]. On the other hand, the three-layer bandage composed of (1) an initial bandage layer made of a paste-impregnated gauze, (2) a compression elastic bandage, (3) a third tubular bandage to support the previously applied two layers [15]. Nevertheless, different types of bandages were reported. A primitive form of paste bandage was developed by Baynton in 1799 [22]. Moreover, in nineteenth century, Unna (a German dermatologist) constructed a rigid bandage made of a rigid plaster incorporated with a woven elastic dressing [23]. It is a short stretch compression bandage made of an initial layer of gauze-impregnated with zinc oxide, calamine lotion, glycerin, gelatin, sorbitol, and magnesium/aluminum compound. In addition to an external elastic wrapping layer producing a graduated compression [24]. Furthermore, a modified Unna boat was also created for advanced venous leg ulcers using only 40% zinc oxide and 60% glycerin past [14]. Furthermore, a single and two-component bandage system is also reported [25].

### Pressure device, sites, positions, levels, and measurements of the sub-bandage pressure

Currently, there are a wide range of commercially available devices and sensors which may be used to quantify interface pressure applied by compression bandages. For measuring pressure interface, all sensors are placed between the supporting surface and the wrapped compression bandage. In the current study, the sub-bandage pressure was measured by a device called medical stocking tester [MST] (Salzmann; Switzerland) [26]. These devices nowadays, acquired the brand name medical stocking tester (MST) have become essential aids to quality control in various textile laboratories all over the globe. In the current study we used the simplest multi-pressure tester (MST MPT-4/MPT-7 [SWISSLASTIC AG ST GALLEN], Switzerland) with the latest addition to the family. The (MST MPT-4/MPT-7) pressures can be measured simultaneously at different positions. The principle of MST measurement is based on the utilization of a pressure transducer. The commonly used unit of compression pressure is the mmHg. In compression garments, (1 mmHg = 133.322 Pascal) is used to define the pressure value [27, 28]. Moreover, the sub-bandage pressure is distributed to be measured at the following points. The dorsum of the foot, two cm above the medial malleolus, in the gaiter area, at the mid-calf, below the knee, at the pre-tibial area, and at the tendo-Achilles. We chose a pressure interface of 35 to 45 mmHg (sitting position, medial gaiter area) to examine different multilayer bandage systems at rest and during exercise. A pressure interface of 40 mmHg at the medial gaiter area is clinically effective and generally accepted and still tolerated by most of the patients [29]. We may consider that the lower limb is a dynamic system. When applying compression to the limb its shape will be changed with muscle contraction. A stiff compression generates an elevated intermittent (working) interface pressures and a constant (resting) pressure [30]. When considering the elastic compression bandage, the first inner layer acts by keeping the ulcer dressing in place and is removed only during dressing change. It may be worn during the day and night, as the sustained low pressure of 20 mmHg is well-tolerated, even in the supine position. The second layer exerts pressure of 20–25 mmHg. It is wrapped on top of the inner layer. The complete kit exerts a pressure of around 40 mmHg in the supine position, rising to almost 50 mmHg in the standing position, which is useful to promote CVLU healing. However, elastic characteristics of a compression system may be indicated by the “static stiffness index” (SSI). Which can be measured by calculating the difference between the working (i.e., standing, walking and exercise), and the resting (i.e., supine) pressures (in mmHg). The SSI of inelastic material is almost always more than 10 mmHg, while elastic bandage shows an SSI less than 10 mmHg. The SSI of the four-layer bandage appeared to be in the range of inelastic material (i.e., more than 10 mmHg). For example, if the supine pressure is 40 mmHg and the standing pressure increases to 55 mmHg, the SSI is 15 mmHg. This SSI is measured by recording the pressure at the interface between the multilayer compression system and the skin (i.e., the interface pressure). Furthermore, we have two methods for sub-bandage pressure measurement (i.e., the supine and the standing pressure). In the supine position, the measurement of pressure exerted on the leg is taken, while the leg is within heart level, while the patient lays down with relaxed knee and ankle joints. While in the standing position the pressure is measured 2–3 min after the patient becomes in this position [31–33].

### RVP medications and technique

The principal of RVP is based on the administration of local intravenous regional anesthesia. Before the RVP procedure begins a manual tourniquet is squeezed over a layer of cotton bad with an Esmarch bandage to completely empty the arterial and venous circulation (Bier's block), of the injected/infused limb followed by the application of a fixed ischemic tourniquet at a high above-knee level. The inflation pressure was set to 20–30 mmHg above the systolic blood pressure to obstruct both venous and arterial circulation [34]. At this point the tourniquet becomes an ischemic tourniquet. To achieve better therapeutic effects the blockage of arterial and venous circulation took place for 30 min [35]. The Esmarch bandage was then removed from the distal (foot) to the proximal (mid-thigh) region to expose the limb and the previously inserted standard venous cannula/CVC. In each RVP session, 80 ml of pharmacotherapeutic agents were sequentially injected via four plastic syringes each containing 20 ml. Followed by consecutive injection of 100 ml of NaCl 0.9% solution. The RVP procedure started with the administration of, (1) a combination of the commercially available Lidocaine HCL 2% (Xylocaine, AstraZeneca) at a dosage of 3 mg/kg [the pH of the unbuffered Lidocaine solution was 5.4 ± 1.1, whereas the pH for the buffered Lidocaine was 7.3 ± 0.2]. The 2nd added component was 8.4% sodium bicarbonate (NaHCO_3_), with a dilution ratio of 10:1 (i.e., 0.5 ml NaHCO_3_ added to 5 ml Lidocaine) [36]. A small volume of normal saline (NaCl) 0.9% solution was added to the previously mixed therapy and freshly buffered solution was made to complete 20 ml of mixed Lidocaine and NaHCO_3_; (2) unfractionated heparin (UFH) 5000 IU/ml was diluted with 19 ml of NaCl 0.9% to complete another 20 ml volume therapy; (3) alprostadil (Prostaglandin E_1_) [37], (Prostavasin, Schwarz Pharma, Monhcim, Germany) was given at 60 mg/session dissolved in 600 ml of NaHCO_3_ 0.9%, given by intravenous infusion at a rate of 200 ml/h/session. Finally, (4) five 20 ml (100 ml) plastic syringes filled with 0.9% NaCl solution were used. Following completion of the RVP therapeutic sessions. As long as the tourniquet is inflated, the RVP therapeutic agents are still in the injected/infused limb, and most of the therapeutic materials have been absorbed into the tissues within 15–20 min. The ischemic tourniquet was left in place for 30 min and then gradually deflated.

### Follow-up

All patients were followed every 15 days after complete ulcer healing for a maximum of 90 days. This was carried out according to the guidelines proposed by the Food and Drug Administration (FDA) and the European Wound Management Association [38]. The following parameters were evaluated, and ulcer pain was assessed on a 10 cm visual analog scale from 0 to 10, where 0 = no pain, and 10 = maximum pain. The existence of serious or fibrinous discharge was classified as (complete, medium, none), the presence or absence of either necrotic or granulation tissues was also reported. In addition to participant satisfaction, as estimated by the VCSS and HRQOLS.

### Important definitions

Intractable venous ulcers were defined as ulcers lasting > 6 weeks, resistant, long-standing venous leg ulcers ≥ 3 cm^2^ that did not respond to conservative therapy. Complete ulcer healing was defined as a full re-epithelialization of the wound with an absence of secretion. Ulcer recurrence was defined as breakdown of an epithelized healed wound. However, the healing rate may be calculated using the following formula, according to Blecken et al., [39]: healing rate = (initial ulcer area–final ulcer area)/number of weeks taken for complete ulcer healing.

### Study endpoints

The primary endpoints were improvement in the ulcer area as assessed by the following: (1) relative reduction in ulcer size. Complete granulation and epithelialization of the wound occurred during RVP sessions and achieving complete ulcer healing was achieved whenever possible by the end of the proposed RVP sessions. This may be defined as complete epithelialization of the wound without scab (eschar) with no need for further wound dressing/compression. The time to healing was assessed from the first day of the procedure until the first date of reduction in ulcer size and consequently complete ulcer healing. The secondary endpoint was patient satisfaction, which was evident from improvement in the patient’s quality of life. This can be measured by improving both VCSS and HRQOLS.

## Statistical analysis

The obtained data were statistically analyzed using the Statistical Package for the Social Science (SPSS®) software program, version 26 (IMB Corporation, Armonk, NY, USA). Continuous variables are described as the means ± standard deviations (SDs), whereas categorical variables are expressed as percentages. On the other hand, for categorical variables, such as sex, nonhealing ulcers, and recurrence rate, we used the chi-square test (χ^2^) to compare differences between the two groups. Survival functions from ulcer recurrence were analyzed via Kaplan–Meier survival analysis to estimate the percentage of patients who experienced complete ulcer healing, and patients who survived without mortality, recurrence, or delayed ulcer healing. Groups were compared using the log-rank test. However, the log-rank test was performed to assess if curves differ significantly or not. Furthermore, unadjusted hazard ratios with 95% confidence intervals (CIs) were calculated to estimate the association between risk factors and the development of chronic venous leg ulcers. The results are expressed as *p* values. Statistical significance was considered when *p* was < 0.05.

## Results

During a 4-year retrospective study period, 384 patients with lower extremity intractable CVLUs were analyzed. All treated patients had chronic resistant nonhealing venous leg ulcerations with variable diameters and durations. The patients'demographic characteristics and the CEAP stratification for the treated groups are listed in Table 1. Patients’ comorbidities and the causes of the development of intractable venous ulceration are reported in Table 2. However, ulcer duration, the number of RVP sessions, and the mean healing/reduction in ulcer time are displayed in Table 3. There were statistically significant correlations between the mean ulcer surface area and the number of therapeutic RVP sessions, evident by the reduction in the ulcer surface area (*p* = 0.001 and 0.003, respectively), as reported in Table 4.

**Table 1:** Patients'demographic characteristics and CEAP classifications of the treated groups

Factor	RVP treated group (Group I)								Compression bandage group (Group II)							
Age	***n***	**Mean**		**SD**	**Range**	**Min**		**Max**	***n***	**Mean**		**SD**	**Range**	**Min**		**Max**
	192	37.4		5	30	19		49	192	40.2		6.1	27	25		52
	The overall median age of both treated and control groups (group I and group II) were 37.26 ± 4.2 (range: 27–43) years															
Sex	**Female**				**Male**				**Female**				**Male**			
	***n***		**%**		***n***		**%**		***n***		**%**		***n***		**%**	
	132		69		60		31		115		60		77		40	
	The overall total number of the studied groups (group I and group II) were summarized as follows: the total number of both groups was 384 patients (192 treated with innovative RVP therapy [group I]). While the number of the control group (group II) was also (192 patients). They are collectively stratified as follows 75% (n = 288) females and 25% males (n = 96), with the female to male ratio 3:1															
CEAP classifications of the RVP treated group																
C_2_,_S_, E_p_, A_s_, P_r_	Varicose veins															
C_3_,_S_, E_p_, A_s_, P_r_	Edema															
C_6_,_S_, E_p_, A_s_, P_r_	Active venous ulcer															
C_6r_,_S_, E_p_, A_s_, P_r_	Recurrent active venous ulcer															

CVLUs, Chronic Venous Leg Ulcers; SFJ, Saphenofemoral Junction; SPJ, Saphenopopliteal Junction; RVP, Retrograde Venous Perfusion; SD, Standard Deviation; n, number; Min, Minimum; Max, Maximum; CEAP classification, Clinical Etiological Anatomical Pathological classification

**Table 2:** Patients'comorbidities and causes of CVLUs for the RVP treated group (Group I) and the control group (compression bandage group [Group II])

Comorbidities	*n*	%
Obesity	63	33
Long standing	81	42
BMI (kg/m^2^)		
Overweight = 25–29.9	32	17
Obese = **>**30	27	14
Dyslipidemia	23	12
Diabetes mellitus	48	25
Recurrent ulceration	200	52
Previous operations due to superficial vein reflux	32	17
Causes of venous leg ulcers		
Saphenofemoral junction (SFJ) incompetence	42	22
Saphenopopliteal junction (SPJ) incompetence	19	10
Superficial vein varicosities with superficial valvular incompetence	48	25
Perforator incompetence	24	12.5
Post-thrombotic ulcer	80	41.5
Total	**192**	**100**

CVLUs, Chronic Venous Leg Ulcers; SFJ, Saphenofemoral Junction; SPJ, Saphenopopliteal Junction; RVP, Retrograde Venous Perfusion; BMI, Body Mass Index

**Table 3:** Ulcer surface area and size for group I (RVP treated group), measured by the Kundin method formula and the ruler-based measurement for circumferential ulcer, respectively, its duration, and the number of RVP sessions

Group I (RVP treated group)						
Factor	No	Mean	± SD	Range	Min	Max
Ulcer surface area in cm^2^						
Kundin method formula	135	19.2 cm^2^	16.8	43.9 cm^2^	15.7 cm^2^	43.9 cm^2^
Ulcer size						
Ruler-based measurement for circumferential ulcer	57	197.9	28.3	67	175.5	242.5
Ulcer duration (in weeks)	192	23.04	12.4	42	6	48
Number of RVP sessions (in weeks)	192	26.78	7.6	24	12	36
Healing time (in days)	192	76.95	28.5	75	45	120

RVP, retrograde venous perfusion; SD, standard deviation; Max, maximum; Min, minimum; GI, Group I; cm^2^, centimeter square

**Table 4:** Correlation between the number of RVP sessions and the reduction in the surface area in cm^2^ of the treated venous leg ulcers measured by the Kundin method formula

	Sum of Squares	df	Mean Square	F	Sig
Ulcer Surface Area in cm^2^ measured by the Kundin method formula					
Between groups	8834.142	3	2944.714	69.422	0.001^*^
Within groups	5556.696	131	42.418		
Total	14,390.837	134			
Circumferential ulcers measured by the rural-based method					
Between groups	2084.481	2	1042.241	77.248	0.003^*^
Within groups	728.571	54	13.492		
Total	2813.053	56			

RVP, retrograde venous perfusion; cm^2^, centimeter square; df, degree of freedom; F, for the one-way ANOVA test; ^*^ Significant

**Fig. 3:**
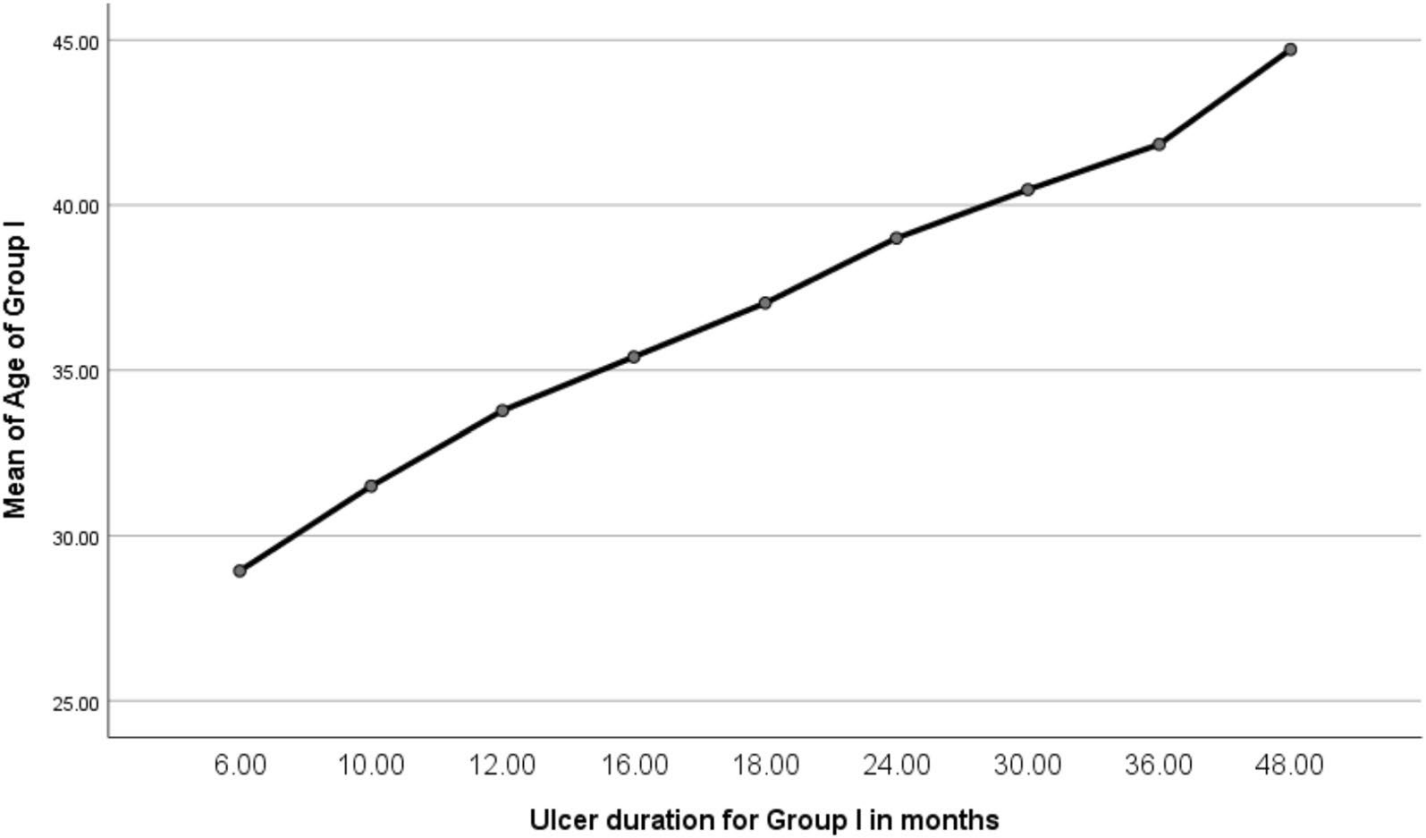
Positive correlation between the patient’s ages in the RVP treated group and the ulcer duration

For Group II, the ulcer size and duration are listed in Table 5. Superficial thrombophlebitis was reported in 15% (*n* = 29) of patients at the site of peripheral cannulation, in addition to central venous cannula, nevertheless, 3% (*n* = 6) developed cardiac arrest as a result of sudden release of the tourniquet during RVP sessions. Furthermore, there was a significant correlation between the patient age in the RVP treated group and the ulcer duration (*p* = 0.001), as shown in Fig. 3, and reported in Table 6.

**Table 5:** Ulcer surface area and its size for group II (bandage compression group), measured by the Kundin method formula and the ruler-based measurement for circumferential ulcers, respectively, and their duration

Group II (Compression Bandage Group)						
Factor	no	Mean	± SD	Range	Min	Max
Ulcer Surface Area in cm^2^ measured by the Kundin method formula						
Kundin method formula	124	30 cm^2^	11.3	29.8 cm^2^	15.7 cm^2^	49.9 cm^2^
Ulcer size measured by rural-based method						
Ruler-based measurement for circumferential ulcers	68	158	18.3	46.6	139	185
Ulcer duration (weeks)	192	33.4	10	35	8	43

SD, standard deviation; centimeter square, cm^2^; no, number; Max, maximum; Min, minimum

**Table 6:** There was a statistically significant correlation between the patient’s age of the RVP treated group and the ulcer duration using the one-way ANOVA test

	Sum of Square	df	Mean Square	F	Sig
Between groups	3855.789	8	481.974	92.484	0.001^*^
Within groups	953.690	183	5.211		
Total	4809.479	191			

RVP, retrograde venous perfusion; df, degree of freedom; F, for one-way ANOVA test; ^*^ significant

In addition, there was a statistically significant difference in the ulcer size before and after the RVP sessions, compared with the paired samples *t* test (*p* = 0.001), as reported in Table 7. However, we achieved a 96.8% (*n* = 186) reduction in ulcer size within a period ranging between 45 and 120 days, where 18 to 36 RVP sessions were performed (Table 8).

**Table 7:** Paired samples *t* test comparing the ulcer size before and after different RVP therapeutic sessions

	Paired samples *t* test—paired differences								
			SD	SE	95% confidence interval of the difference		*t*	*df*	Sig (two-tailed)
		Mean		Mean	Lower	Upper			
Pair 1	Ulcer size before RVP -Ulcer size after 18 RVP sessions with reduction in ulcer size in 35% (n = 67) of patients	27.61940	5.88702	0.71921	26.18345	29.05536	38.402	66	0.001^*^
Pair 2	Ulcer size before RVP -Ulcer size after 24 RVP sessions with reduction in ulcer size in 10.5% (n = 21) of patients	22.67895	1.63353	0.37476	21.89161	23.46628	60.516	18	0.001^*^
Pair 3	Ulcer size before RVP—Ulcer size after 28 RVP sessions with reduction in ulcer size in 12.5% (n = 24) of patients	22.03750	1.92869	0.39369	21.22309	22.85191	55.977	23	0.001^*^
Pair 4	Ulcer size before RVP—Ulcer size after 32 RVP sessions with reduction in ulcer size in 18% (n = 34) of patients	22.49412	3.79009	0.65000	21.17169	23.81654	34.607	33	0.001^*^
Pair 5	Ulcer size before RVP—Ulcer size after 34 RVP sessions with reduction in ulcer size in 9% (n = 17) of patients	23.25625	0.97500	0.24375	22.73671	23.77579	95.410	15	0.001^*^
Pair 6	Ulcer size before RVP—Ulcer size after 36 RVP sessions with reduction in ulcer size in 15% (n = 29) of patients	21.61724	1.98334	0.36830	20.86282	22.37166	58.695	28	0.001^*^

^*^Significant

**Table 8:** Reduction in ulcer size at different study periods following the administration of RVP sessions and the number of RVP sessions for each study period according to the ulcer size and its response to pharmacological agents

Duration of ulcer healing	No. of RVP sessions	*n*	%
Reduction in ulcer size at 45 days	18	67/192	34.8
Reduction in ulcer size at 60 days	24	14/192	7.2
Reduction in ulcer size at 75 days	28	24/192	12.6
Reduction in ulcer size at 90 days	32	36/192	19
Reduction in ulcer size at 105 days	34	16/192	8.3
Reduction in ulcer size at 120 days	36	29/192	15
Total	172	186	96.9

RVP, retrograde venous perfusion; CVLUs, chronic venous leg ulcers; n, number

**Fig. 4:**
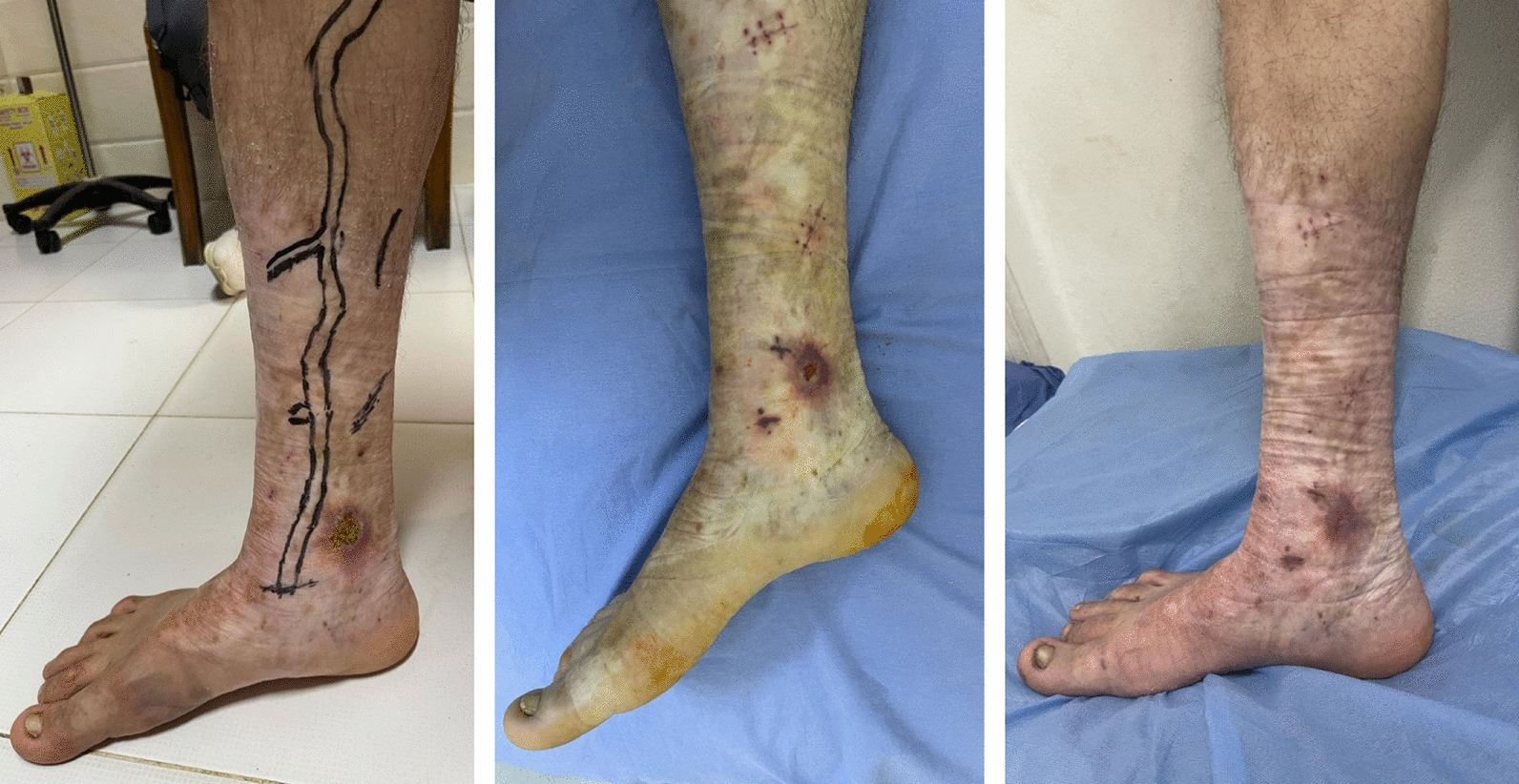
Treatment of below the knee varicosities causing venous leg ulcers, showing complete ulcer healing

Ablation of truncal varicosities and superficial venous reflux were performed in selected patients. Most of our patients were subjected to the proper and convenient treatment modality of superficial varicosities with respect to their preference. The type of ablated superficial vein reflux was chosen according to the anatomical level of vein valve incompetence, as shown in Fig. 4, where below the knee varicosities with incompetent valves were treated.

Moreover, venous intervention was followed by the administration of RVP therapeutic sessions for rapid healing of adjacent venous leg ulcer. All operated patients received a three-layer compression bandage with a pressure of 50–60 mmHg in the sitting position. While a pressure of 70 mmHg was applied in the standing position [40].

**Fig. 5:**
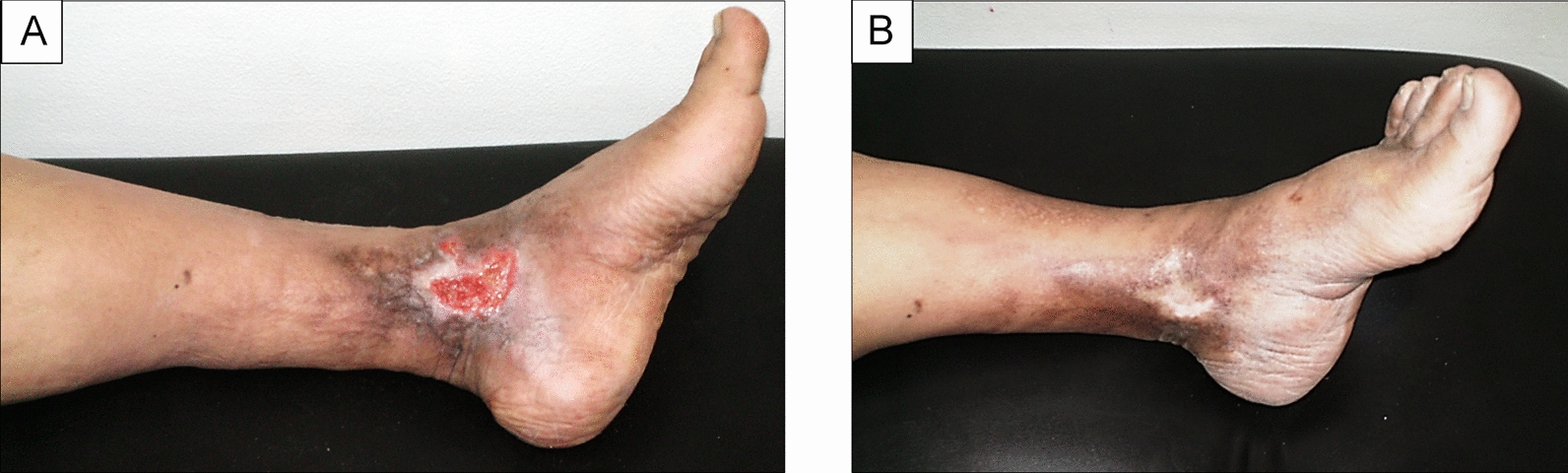
Post-thrombotic intractable venous leg ulcer before (**A**); and following RVP sessions, showing complete healing after 75 days (**B**)

**Fig. 6:**
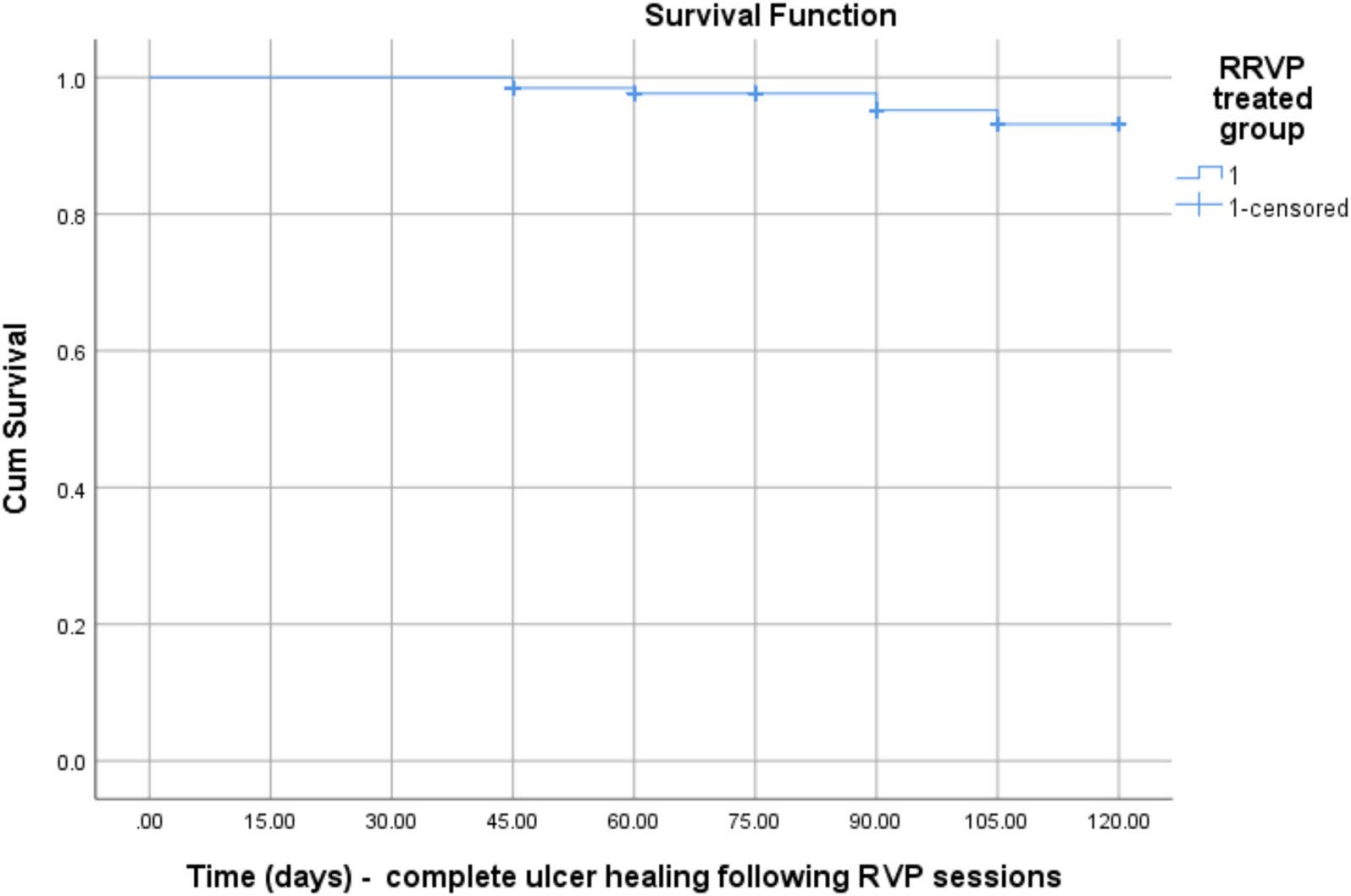
Kaplan–Meier curve demonstrates complete healing of CVLUs following the innovative RVP procedure within a calculated period ranged between 45 and 120 days

During the follow-up period, a progressive reduction in ulcer size was observed in both groups. Nevertheless, in the GI, a faster reduction in wound size was observed within a short period of time. However, complete ulcer healing (Fig. 5A, B), was observed in most patients and ranged between 54 to 120 days after RVP sessions as determined by the Kaplan–Meier survival analysis (Fig. 6). Furthermore, there was a highly significant difference between the RVP treated group (*p* = 0.007), and the control group (*p* = 0.468) as reported in Table 9 using the one-way ANOVA test.

**Table 9:** One-way ANOVA test comparing between GI and GII

	ANOVA table					
		Sum of Square	df	Mean Square	*F*	Sig
RVP treated group (group I)	Between groups	1.782	1	1.782	7.227	0.007^*^
	Within groups	94.218	382	0.247		
	Total	96.000	383			
Compression bandage group (group II)	Between groups	433.753	1	433.753	0.529	0.468^∳^
	Within groups	155,787.992	190	819.937		
	Total	156,221.745	191			

RVP, retrograde venous perfusion; df, degree of freedom; F, for one-way ANOVA test; ^*^significant; ^∳^ non-significant

**Fig. 7:**
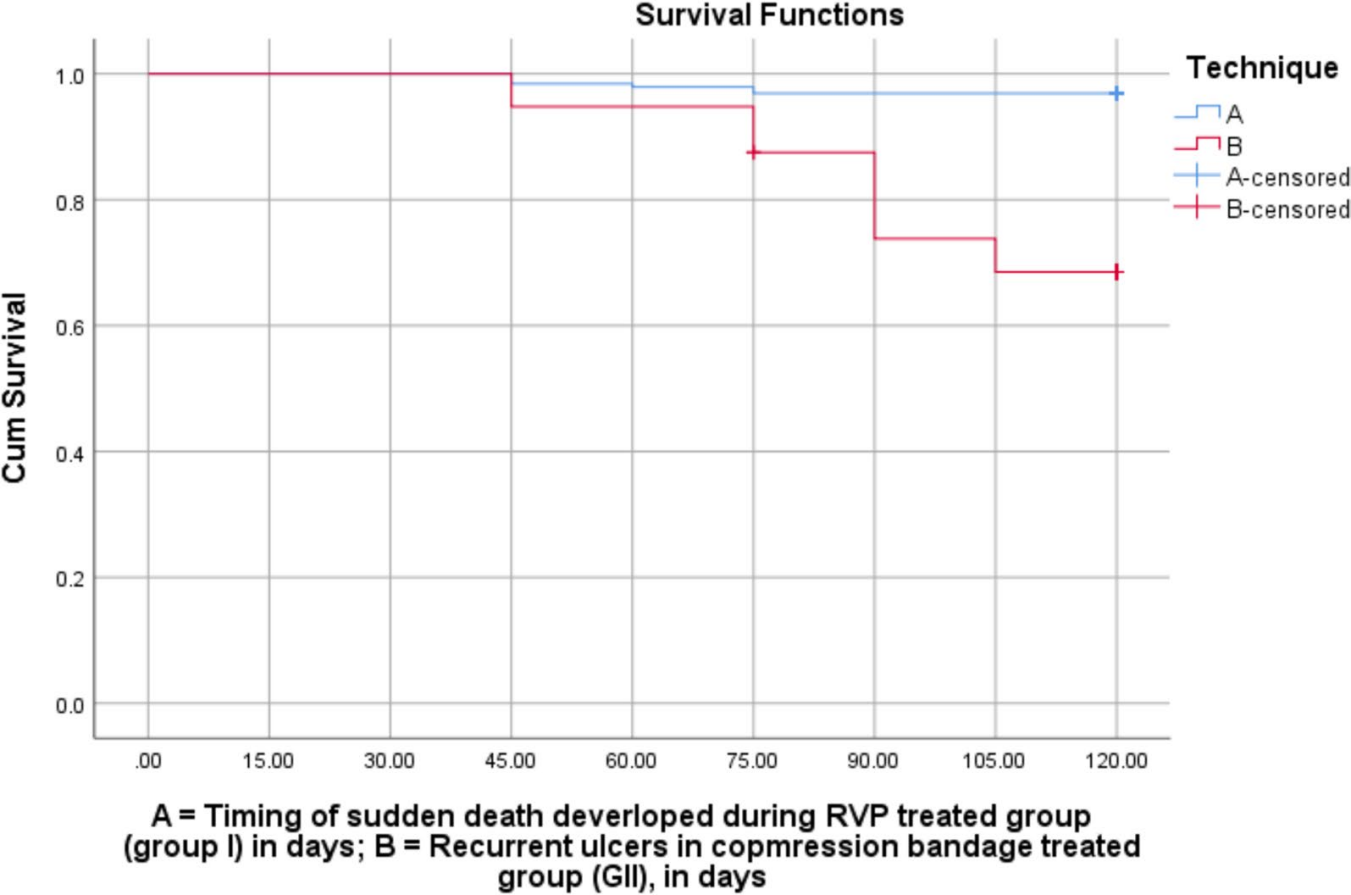
Test of equality of survival distributions between group I (RVP group) and group II (control group), using the Kaplan–Meier survival curves

Moreover, we used test of equality of survival distribution for the different levels of technique using the Kaplan–Meier survival analysis, to demonstrate patients free from complications, mortality, and ulcer recurrence/nonhealing in the RVP treated group compared to the compression bandage group, as shown in Fig. 7), in addition to the log-rank test (*p* = 0.001), as reported in Table 10. However, following the administration of RVP therapeutic agents in combination with compression bandage technique and local wound care, there were marked improvements in both VCSS and HRQOLS (*p* = 0.001 and 0.001), respectively, as reported in Table 11. The results of this cohort study indicated that there is high-quality evidence of the safety and effectiveness of innovative RVP therapy for the healing of intractable chronic venous ulcer leg ulcers. Furthermore, after completing a 12-month follow-up period, the rate of ulcer reduction/healing was achieved in 96.9% (*n* = 186/192) for the RVP therapeutic group and 68.8% (*n* = 132/192) for the control (compression bandage) group.

**Table 10:** Test of equality of survival distributions for the different levels of technique

	Chi-square	df	Sig
Log rank (Mantel–Cox)	52.257	1	0.001^*^
Breslow (generalized Wilcoxon)	50.695	1	0.001^*^
Tarone-Ware	51.506	1	0.001^*^

^*^Significant

**Table 11:** Paired samples *t* test comparing both VCSS and HRQOLS before and after RVP procedure

	Paired samples *t* test—paired differences				95% confidence interval of the difference		t	df	Sig (two-tailed)
		Mean	SD	SE Mean	Lower	Upper			
Pair 1	VCSS before RVP therapy—VCSS after RVP therapy	1.99479	0.07217	0.00521	1.98452	2.00506	383.000	191	0.001^*^
Pair 2	HRQOLS before RVP therapy—HRQOLS after RVP therapy	1.96785	0.17445	0.01259	1.94392	1.99358	156.378	191	0.001^*^

RVP, regional retrograde venous perfusion; VCSS, Venous Clinical Severity Score; HRQOLS, Health-Related Quality-of-Life Score; SD, standard deviation; SE, standard error; df, degree of freedom; ^*^ significant

## Discussion

Resistant long-standing CVLUs are a challenging problem associated with significant morbidity, and functional disability, with considerable changes in the patient’s life quality. However, the real mechanism behind the development of intractable chronic venous ulcers may be attributed to “venous hypoxia” resulting from hypervolemia of deoxygenated venous blood which leads to tissue ischemia and, eventually, venous leg ulcers. The treatment of these ulcers should be a multistage process, in which patient management may take two main directions. Conservative management and interventional treatment in selected cases. Moreover, graduated compression therapy may not result in proper and sustained wound healing. However, to date there is no clinical evidence of the use of adjuncts or other successful alternatives to improve outcomes [41]. In the current study, we reported our initial experiences with the safety and effectiveness of a new therapy for the management of CVLUs in comparison with the conventional compression bandage technique. This therapeutic technique is known as the retrograde venous perfusion (RVP) procedure. It comprises regional administration of various drug therapies as an adjuvant treatment of both local wound dressing and compression therapy of the diseased limb. The pharmacotherapeutic drugs involved in the RVP sessions included 2% Lidocaine Hcl at a dosage of 3 mg/kg combined with 8.4% NaHCO3 (1 mec/10 ml), so 2 mec was completed to 20 ml 0.9% sodium chloride solution [42]. Lidocaine is the prototypical local anesthetic and was identified as the first sodium channel blocker. It acts through blocking K^+^ and Na^+^ and ion channels and regulates extra- intracellular calcium concentrations through other ligand-gated ion channels. Its main mechanism of action is blocking voltage-gated Na^+^ channels (VGSC/NaVs). This regulates the concentration of cell ions, both inside and outside, changes the transmembrane potential, regulates the excitability of neurons, and affects the discharge frequency and action potential conduction speed of nerve fibers [43]. In addition, Lidocaine targets GPCRs and participates in many cell signal transduction processes, which not only explains the mechanism of its analgesic and antihyperalgesic effects but also may explain some of the other clinical effects of Lidocaine, such as its neuroprotective, anti-inflammatory, and anticancer drug sensitization effects [44]. Lidocaine must be alkalinized to prolong its anesthetic effect and shorten the onset of local anesthetics. The cytoplasm of nerve fibers is more acidic than the extracellular fluid (pH = 6.9); therefore, ionization rates and consequently, the effectiveness of drug molecules entering the cytoplasm increase. Accordingly, the nonionized form allows the drug to be more effective by ensuring that the drug reaches the target wound area. However, decreasing the environmental pH increases the Lidocaine ionization rate. Solutions of local anesthetic salts in water used in medicine display acidic reactions. These must be neutralized by the tissue fluids to be effective. Adding alkaline substances such as NaHCO_3_ at a concentration of 8.4–2% Lidocaine Hcl increases the local anesthetic effect by increasing the ratio of the nonionized form and allows easier penetration of the drug to the nerve fibers [45]. Moreover, unfractionated heparin (UFH) administration is an essential component of this technique. In chronic venous ulcers/wounds, there is a deficiency and dysfunction of the extracellular matrix (ECM) which cannot support the healing process. Heparin is a regulator of ECM proteins and consequently helps promote wound healing. Heparin and growth factors have rapid and effective mechanisms in wound healing through their role in prompt and efficient endothelial cell repair. However, UFH continues to have therapeutic advantages for wound healing in CVLUs [46]. Furthermore, alprostadil [prostaglandin E_1_ (PGE_1_)] was added to the therapeutic RVP regimen. PGE_1_ is a naturally secreted prostaglandin that may result in improved blood flow. In the vascular medical field, its use is limited to the treatment of erectile dysfunction, healing of ischemic leg or foot ulcers, and management of critically ischemic limbs [47]. The mode of action of PGE_1_ is not fully understood in reported clinical trials. However, possible actions are believed to be due to vasodilatation of small blood vessels, increased fibrinolytic activity, blood flow augmentation in the capillary network, stabilization of the endothelial membrane, reduction in white cell activation, inhibition of platelet aggregation, inhibition of smooth muscle cell proliferation in the tunica media, and promotion of the development of collateral circulation [48]. Moreover, PGE_1_ has a potent direct vasodilator effect on peripheral vascular beds indirectly through reflex sympathetic stimulation, alterations to cutaneous trophism, and the activation of fibrinogenesis. Moreover, Rudofski [49] believed that this effect may be related to the local improvements in microcirculation. All of these drugs are infused/injected into the lower extremity which has been exsanguinated by compression and has been isolated from the systemic circulation by means of an ischemic tourniquet. Compared with males, females generally have an increased rate of CVLUs, because of obesity, and immobility, and may have a congenital weakness/absence of veins, or a history of phlebitis or deep vein thrombosis (DVT) [50, 51]. Furthermore, our patients’ population were predominantly female rather than male which is in agreement with the findings reported in the literature. We reported 75% (n = 288) female and 25% male (*n* = 96), with a female to male ratio of 3:1, as females frequently asked for medical consultation, which may result in their dominance. Moreover, we reported a median age of 37.26 ± 4.2 years, contradicting the literature report [52, 53], where the patient’s median age was 83 years. However, a study in Egypt reported the age distribution of younger age groups and investigated the impact of modifying occupational risk factors on the outcome of CVLUs. They reported a median age of 39.7 ± 6.3 years and 43.2 ± 8.2 years in both groups [54]. The discrepancy in age distribution between developed and developing countries may be attributed to low socioeconomic status, bad hygienic conditions of the leg, and the negligence of wound cleaning with lack and the negligence of medical consultation because of indigence and poverty. Most importantly the predominance of post-phlebetic etiology, which represents the major number of patients presented with CVLUs as reported previously in Table 1. We choose to treat only patients with C_2_,_S_, E_p_, A_s_, P_r_, (varicose veins), C_3_,_S_, E_p_, A_s_, P_r_ (edema), C_6_,_S_, E_p_, A_s_, P_r,_ (active venous ulcer), and C_6r_,_S_, E_p_, A_s_, P_r_ (recurrent active venous ulcer) of the CEAP stratification, which may be in agreement with that reported in the previous literature [55]. In the current study, many risk factors and comorbidities were reported to play a role in development, recurrence, and resistant ulcer healing, which coincides with that reported in previous literature reports [56–58]. They described their role in delayed healing time with resistant venous ulcers, contradicting that reported in the current study, where a reduction in ulcer size/healing was reported within a short period after RVP medication. A previous literature report introduced other innovative methods and techniques for CVLUs. However, these new therapies and technologies differ from those reported in this study. They applied biophysical therapy using ultrasound, electromagnetic therapy, electrical stimulation, and phototherapy. Moreover, platelet-rich plasma (PRP) injection therapy, stem cell therapies, biologic skin equivalents, oxygen therapies, anti-TNF therapy, or negative pressure wound therapy, are advanced venous ulcer therapeutic methods that may support the standard of care. Moreover, medical devices, such as a muscle pump activator, or intermittent pneumatic compression device, may be especially useful for specific subgroups of patients suffering from CVLUs. However, they concluded that the treatment of CVLUs with innovative therapies, techniques, and technologies still requires large, high-quality, randomized controlled clinical trials (RCCT) [59]. Other literature reported the value of systemic delivery of hyperbaric oxygen as an adjunctive to venous intervention for management of non-healing resistant venous leg ulcers, contradicting our novel RVP procedure. These results are in accordance with those of the current study, where they reported the significant value of this combination therapy in the process of rapid healing of resistant long-standing venous leg ulcerations within a short period of time [60]. Furthermore, a recently published article reported the use of 80 mg of Ixekizumab, (a monoclonal antibody, that inhibits IL17 A). They suggested that antagonizing IL17 might accelerate wound healing by reducing the wound size from an average of 955 to 529 mm^2^ at week 0 to week 14, contradicting our use of drug therapy which involves a combination of Lidocaine, NaHCO_3_, UFH, and alprostadil, that may be available in our resource-challenged environment. They achieved good results in terms of reducing ulcer size within 14 weeks. Moreover, they reported that this marked improvement in ulcer healing in response to high dose of Ixekizumab injection accelerated healing supporting the genetic deletion of IL17/anti-IL17 therapy [61]. However, the use of a combination of many therapeutic agents including combined local physical therapy with the device Laserobaria-S, including simultaneous action of an extremely low-frequency variable magnetic field, hyperbaric oxygen, and low-energy light radiation was reported in the literature [62]. This combination therapy contradicts our RVP pharmacotherapeutic procedure. Nevertheless, they achieved 100% wound healing with 9 weeks of therapeutic cycles performed every day for 30 days. Their results are superior to our results, where complete ulcer healing was reported in the first 45 days of treatment in some patients. The best healing results were achieved in the group in which 18 RVP sessions were used. Physiologically, acute wounds heal within 4 weeks of treatment. Moreover, chronic wounds like CVLUs need a longer time to heal, with a healing time range between six to 12 months. Furthermore, recurrence was reported in 70% of CVLUs within 5 years of complete ulcer healing [63]. However, we have several factors that may delay or prolong the time of venous ulcer healing. These factors include advanced age, obesity with increased body mass index (BMI > 25 kg/m^2^), and nutritional deficiencies, history of DVT, inadequate compression therapy, and larger ulcer surface area and long ulcer duration. Furthermore, locally infected ulcers, active ulcers, and the use of antibiotics, all these factors are considered poor prognostic signs for ulcer healing. Moreover, the history of prior ulceration may be considered a potential risk for poor/delayed ulcer healing. In addition, if the ulcer floor contains more than 50% fibrinous exudate, it may be associated with delayed/resistant healing. These situations are very common and may be considered improper or ineffective prior attempts at the start of therapy [3, 64]. The longer the ulcer activity, the difficult to achieve ulcer healing. Time-dependent changes may develop in the ulcer microenvironment. These changes include extreme production of collagenases, elastases, and matrix metalloproteinases. This leads to a rapid breakdown of growth factors and collagen. In addition to the phenotypic variations of the wound cells, especially fibroblasts, which may prevent their ability to proliferate and move. Moreover, a hypoxic microenvironment may contribute to a high rate of fibroblast proliferation, leading to tissue fibrosis, as well as a high susceptibility for fungal and bacterial colonization [65]. Many literature reports that colonization and infection have a pivotal role in delaying the healing process of venous ulcers [66, 67]. According to the previous evidence, we may suggest that further RVP sessions were used in individual groups; the healing time will not be the same for the same number of patients and may differ from one group to another. Furthermore, the tissues may take a longer time to regenerate, and additional RVP sessions may be needed to effectively regenerate granulation tissue and promote the healing process of CVLUs. Finally, the use of innovative RVP therapy may accelerate the healing process of CVLUs in addition to both local ulcers dressing and the conventional multilayer compression bandage technique. Moreover, current evidence supporting the use of the RVP injection/infusion technique is currently sufficient, as documented by our previously reported results, indicating increased blood flow after RVP to the target area. However, a significant long-term improvement was observed with RVP pharmacotherapy up to 6 months after the end of therapy. Furthermore, the favorable effects of this therapeutic regimen may be explained by alterations in endothelial function caused by repeated limb occlusions and hemodynamic changes in the periulcer network. This could provide some way toward addressing the significant improvement of VCSS and HRQOLS associated with CVLUs. Furthermore, the role of the RVP technique has not been published in the previous literature reports for the treatment of non-venous leg ulcers and these results may suggest that the RVP technique has a good benefit for the treated limb. However, its relative safety and efficacy may suggest that it may be optimal treatment for all patients with resistant CVLUs. The present study confirms the satisfactory results with respect to a decrease in ulcer size, overcoming critical complications that may threaten the limb, increasing blood flow to the wound area, and confirming the progression of wound healing and conserving the foot to avoid deformities.

### Prevention of ulcer recurrence

The recurrence rate of CVLUs has been reported to be as high as 60–70% after 10 years. Venous intervention and long-term application of elastic compression stockings are essential factors for preventing ulcer recurrence. Limb elevation may be favorable when used with compression stockings, and calf muscle pump function may be improved by encouraging exercise. Moreover, self-efficacy and proper socioeconomic support may play a role in preventing venous leg ulcer recurrence [68, 69].

### Study strengths and limitations

To the best of our knowledge, no review article has published an adjuvant treatment to compression therapy for management of intractable venous leg ulcers. However, the current study may be the first innovative and new therapeutic option reported in the literature, including a timely installation of high-quality evidence compared with the previous literature reports which used repeated, traditional methods and conventional therapeutic procedures for treating intractable venous leg ulcers, with poor results. Moreover, this study has several limitations. There are no available data or guidelines that distinguish patients who will undergo this therapeutic technique. The available guidelines only mentioned compression therapy in addition to the interventional venous procedures (2022, ESVS Guidelines). Another limitation is the retrospective nature of this work. Therefore, further studies still require large, high-quality, randomized controlled clinical trials. Moreover, the cost-effectiveness and long-term follow-up of various pharmacological agents used in RVP therapeutic procedure as well as the timing of ulcer healing must be addressed in future research.

## Conclusions

Retrograde venous perfusion is a pharmacotherapeutic procedure based on regional intravenous anesthesia. It was adopted to treat intractable venous leg ulcers. It may be a safe, beneficial, and effective technique as an adjunctive to local wound dressing and compression therapy. It may represent a promising tool for patients with intractable venous leg ulcers and provide essential evidence to reduce the negative social and economic impact.

## Data Availability

No datasets were generated or analysed during the current study.

## Abbreviations

ABI: Ankle/Brachial Pressure Index
BMI: Body Mass Index
CEAP: Clinical, Etiological, Anatomical, Pathological
CI: Confidence Interval
CVC: Center Venous Cannula
CVLU: Chronic Venous Leg Ulcers
DVT: Deep Vein Thrombosis
ECM: Extracellular matrix
FDA: Food and Drug Administration
GI: Group I
GII: Group II
GPRS: G Protein-coupled receptors
GSV: Great Saphenous Vein
HRQLS: Health-related quality-of-life score
PAD: Peripheral Arterial Diseases
PGE1: Prostaglandin E_1_
PRP: Platelet-Rich Plasma
RCCT: Randomized Controlled Clinical Trials
RVP: Retrograde Venous Perfusion
SD: Standard Deviation
SSI: Static Stiffness Index
SPSS: Statistical Package for Social Science
UFH: Unfractionated Heparin
VCSS: Venous Clinical Severity Score

## References

[1] Klein A, Ennis W, Fukaya E. Characteristics of venous leg ulcer patients at a tertiary wound care center. J Vasc Surg Venous Lymphat Disord. 2023;11:270-79.e1. 36410701 10.1016/j.jvsv.2022.09.018

[2] Kirsner RS, Vivas AC. Lower-extremity ulcers: diagnosis and management. Br J Dermatol. 2015;173:379–90. 26257052 10.1111/bjd.13953

[3] Scotton MF, Miot HA, Abbade LPF. Factors that influence healing of chronic venous leg ulcers: a retrospective cohort. An Bras Dermatol. 2014;89:414–22. 24937814 10.1590/abd1806-4841.20142687 PMC4056698

[4] Schul MW, Melin MM, Keaton TJ. Venous leg ulcers and prevalence of surgically correctable reflux disease in a national registry. J Vasc Surg Venous Lymphat Disord. 2023;11:511–6. 36681297 10.1016/j.jvsv.2022.11.005

[5] Shankar S, Ayyappan MK, Palani T, et al. Factors associated with health-related quality of life of south indian population with chronic venous leg ulcers - A hospital based pilot study. J Vasc Nurs. 2022;40:162–6. 36435598 10.1016/j.jvn.2022.09.005

[6] Eberhardt RT, Raffetto JD. Chronic venous insufficiency. Circulation. 2014;130:333–46. 25047584 10.1161/CIRCULATIONAHA.113.006898

[7] Barwell JR, Davies CE, Deacon J, et al. Comparison of surgery and compression with compression alone in chronic venous ulceration (ESCHAR study): randomised controlled trial. Lancet. 2004;363:1854–9. 15183623 10.1016/S0140-6736(04)16353-8

[8] Gohel MS, Heatley F, Liu X, et al. A randomized trial of early endovenous ablation in venous ulceration. N Engl J Med. 2018;378:2105–14. 29688123 10.1056/NEJMoa1801214

[9] Lurie F, Passman M, Meisner M, et al. The 2020 update of the CEAP classification system and reporting standards. J Vasc Surg Venous Lymphat Disord. 2020;8:342–52. 32113854 10.1016/j.jvsv.2019.12.075

[10] Öien RF, Håkansson A, Hansen BU, et al. Measuring the size of ulcers by planimetry: a useful method in the clinical setting. J Wound Care. 2002;11:165–8. 12055939 10.12968/jowc.2002.11.5.26399

[11] Goldman RJ, Salcido R. More than one way to measure a wound: an overview of tools and techniques. Adv Skin Wound Care. 2002;15:236–43. 12368715 10.1097/00129334-200209000-00011

[12] Khoo R, Jansen S. The evolving field of wound measurement techniques: a literature review. Wounds. 2016;28:175–81. 27377609

[13] Jayaseelan E, Aithal VV. Pinch skin grafting in non-healing leprous ulcers. Int J Lepr Other Mycobact Dis. 2004;72:139–42. 15301590 10.1489/1544-581X(2004)072<0139:PSGINL>2.0.CO;2

[14] Balaraju DN, Srinivas CR, Mukhi SV. Modified unna boot and pinch grafting for chronic non-healing venous leg ulcer. J Cutan aesthet Surg. 2008;1:25–6. 20300337 10.4103/0974-2077.41155 PMC2840893

[15] Meyer FJ, McGuinness CL, Lagattolla NRF, et al. Randomized clinical trial of three-layer paste and four-layer bandages for venous leg ulcers. Br J Surg. 2003;90:934–40. 12905544 10.1002/bjs.4173

[16] Nelcon EA. 3 layer paste bandages were more effective than 4 layer bandages for healing venous leg ulcers. Evid Based Nurs. 2004;7:21. 14994694 10.1136/ebn.7.1.21

[17] Ashby RL, Gabe R, Ali S, et al. Clinical and cost-effectiveness of compression hosiery versus compression bandages in treatment of venous leg ulcers (Venous leg Ulcer Study IV, VenUS IV): a randomised controlled trial. Lancet. 2014;383:871–9. 24315520 10.1016/S0140-6736(13)62368-5

[18] Harrison MB, Vandenkerkhof EG, Hopman WM, et al. The Canadian Bandaging Trial: Evidence-informed leg ulcer care and the effectiveness of two compression technologies. BMC Nurs. 2011;13(10):20. 10.1186/1472-6955-10-20 PMC3214126 21995267

[19] Scriven JM, Taylor LE, Wood AJ, et al. A prospective randomised trial of four-layer versus short stretch compression bandages for the treatment of venous leg ulcers. Ann R Coll Surg Engl. 1998;80:215–20. 9682649 PMC2503021

[20] Moffatt CJ, Franks PJ, Oldroyd M, et al. Community clinics for leg ulcers and impact on healing. BMJ. 1992;305:1389–92. 1486301 10.1136/bmj.305.6866.1389 PMC1883915

[21] McCollum CN, Ellison DA, Groarke L, et al. Randomised trialcomparing Profore and the original four layer bandage. Proceedings of the Conference of the European Wound Management Association, Milan, London, Macmillan, 8, 1997.

[22] Baynton T. Descriptive Account of a New Method of Treating Old Ulcers of the Legs (2nd ed). Emery and Adams: Bristol, 1799.

[23] Unna PG. Die Histopathologie der Hautkrankheiten. Berlin, Germany: Verlag Hirschwald; 1894.

[24] Pascarella L, Shortell CK. Medical management of venous ulcers. Semin Vasc Surg. 2015;28:21–8. 26358306 10.1053/j.semvascsurg.2015.06.001

[25] O'Meara s, Cullum N, Nelson EA, et al. Compression for venous leg ulcers (Review). Cochrane Database Syst Rev. 2012;11:CD000265. 10.1002/14651858.CD000265.pub3 PMC7068175 23152202

[26] Kokai O, Kilbreath SL, McLaughlin P, et al. The accuracy and precision of interface pressure measuring devices: A systematic review. Phlebology. 2021;36:678–94. 34018859 10.1177/02683555211008061

[27] Medical Compression Hosiery, Quality Assurance RALGZ 387/1. http://www.tagungsmanagement.org/icc/images/stories/PDF/ral_gz_387_englisch.pdf

[28] Jamshaid H, Mishra RK, Ahmed N, et al. Exploration of effects of graduated compression stocking structures on performance properties using principal component analysis: a promising method for simultaneous optimization of properties. Polymers (Basel). 2022;14:2045. 35631928 10.3390/polym14102045 PMC9143032

[29] Hafner J, Botonakis I, Burg G. A comparison of multilayer bandage systems during rest, exercise, and over 2 days of wear time. Arch Dermatol. 2000;136:857–63. 10890987 10.1001/archderm.136.7.857

[30] Mosti G, Partsch H. Compression stockings with a negative pressure gradient have a more pronounced effect on venous pumping function than graduated elastic compression stockings. Eur J Vasc Endovasc Surg. 2011;42:261–6. 21612949 10.1016/j.ejvs.2011.04.023

[31] Partsch H. The static stiffness index: a simple method to assess the elastic property of compression material in vivo. Dermatol Surg. 2005;31:625–30. 15996410 10.1111/j.1524-4725.2005.31604

[32] Mosti G, Mattaliano V, Partsch H. Influence of different materials in multicomponent bandages on pressure and stiffness of the final bandage. Dermatol Surg. 2008;34:631–9. 18261104 10.1111/j.1524-4725.2007.34119.x

[33] Vowden K. Vowden P. How to guide: Effective compression therapy. Wound Essentials; 2012. p. 7.

[34] Hicks RW, Denholm B. Implementing AORN recommended practices for care of patients undergoing pneumatic tourniquet-assisted procedures. Aorn J. 2013;98:383–93. 24075334 10.1016/j.aorn.2013.08.004

[35] Noordin S, McEwen JA, Kragh JF Jr, et al. Surgical tourniquets in orthopaedics. J Bone Joint Surg Am. 2009;91:2958–67. 19952261 10.2106/JBJS.I.00634

[36] Cepeda MS, Tzortzopoulou A, Thackrey M, et al. Adjusting the pH of lidocaine for reducing pain on injection (Review). Cochrane Database Syst Rev. 2010;12:CD006581. 10.1002/14651858.CD006581.pub2 21154371

[37] Milio G, Minà C, Cospite V, et al. Efficacy of the treatment with prostaglandin E-1 in venous ulcers of the lower limbs. J Vasc Surg. 2005;42:304–8. 16102631 10.1016/j.jvs.2005.03.040

[38] FDA Wound Healing Clinical Focus Group. Guidance for industry: chronic cutaneous ulcer and burn wounds-developing products for treatment. Wound Repair Regen. 2001;9:258–68. 11679134 10.1046/j.1524-475x.2001.00258.x

[39] Blecken SR, Villavicencio JL, Kao TC. Comparison of elastic versus nonelastic compression in bilateral venous ulcers: a randomized trial. J Vasc Surg. 2005;42:1150–5. 16376207 10.1016/j.jvs.2005.08.015

[40] De Maeseneer MG, Kakkos SK, Aherne T, et al. Editor’s Choice - European Society for Vascular Surgery (ESVS) 2022 Clinical Practice Guidelines on the Management of Chronic Venous Disease of the Lower Limbs. Eur J Vasc Endovasc Surg. 2022;63:184–267. 35027279 10.1016/j.ejvs.2021.12.024

[41] Youn YJ, Lee J. Varicose veins and chronic venous insufficiency. Korean J Intern Med. 2019;34:269–83. 30360023 10.3904/kjim.2018.230 PMC6406103

[42] Kapusuz O, Argun G, Arikan M, et al. Comparison of the effects of low volume prilocaine and alkalinized prilocaine for the regional intravenous anesthesia technique in hand and wrist surgery. Biomed Res Int. 2014;2014: 725893. 25133177 10.1155/2014/725893 PMC4123591

[43] Yang X, Wei X, Mu Y, et al. A review of the mechanism of the central analgesic effect of lidocaine. Medicine (Baltimore). 2020;99: e19898. 32332666 10.1097/MD.0000000000019898 PMC7440315

[44] Rosenberg PH. 1992 ASRA Lecture. Intravenous regional anesthesia: nerve block by multiple mechanisms. Reg Anesth. 1993;18:1–5. 8448091

[45] Wong K, Strichartz GR, Raymond SA. On the mechanisms of potentiation of local anesthetics by bicarbonate buffer: drug structure-activity studies on isolated peripheral nerve. Anesth Analg. 1993;76:131–43. 8418714 10.1213/00000539-199301000-00023

[46] Galvan L. Effects of heparin on wound healing. J Wound Ostomy Continence Nurs. 1996;23:224–6. 8900676 10.1016/s1071-5754(96)90095-9

[47] Urciuoli R, Cantisani TA, Carlini M, et al. Prostaglandin E1 for treatment of erectile dysfunction. Cochrane Database Syst Rev. 2004;2:CD001784. 10.1002/14651858.CD001784.pub2 15106162

[48] Prasad BR, Naveen GA, Desai SC, et al. From compression to injections: prostaglandins paving a new direction for venous leg ulcer treatment. Indian J Vasc Endovasc Surg. 2019;6:176–81.

[49] Rudofsky G. Intravenous prostaglandin E1 in the treatment of venous ulcers-a double-blind, placebo-controlled trial. Vasa. 1989;28:39–43. 2692199

[50] Millan SB, Gan R, Townsend PE. Venous Ulcers: Diagnosis and Treatment. Am Fam Physician. 2019;100:298–305. 31478635

[51] Mansilha A, Sousa J. Pathophysiological mechanisms of chronic venous disease and implications for venoactive drug therapy. Int J Mol Sci. 2018;19:1669. 29874834 10.3390/ijms19061669 PMC6032391

[52] Pérez MB, López-Casanova P, Lavín RS, et al. Epidemiology of venous leg ulcers in primary health care: Incidence and prevalence in a health centre-A time series study (2010–2014). Int Wound J. 2019;16:256–65. 30393963 10.1111/iwj.13026 PMC7949455

[53] Refaat TM, Ewis AA, Osman AO, et al. The impact of modifying occupational risk factors on the outcome of treatment of chronic venous ulcer. Egypt J Occup Med. 2011;35:277–87.

[54] Obermayer A, Garzon K. Identifying the source of superficial reflux in venous leg ulcers using duplex ultrasound. J Vasc Surg. 2010;52:1255–61. 20692796 10.1016/j.jvs.2010.06.073

[55] Milic DJ, Zivic SS, Bogdanovic DC, et al. Risk factors related to the failure of venous leg ulcers to heal with compression treatment. J Vasc Surg. 2009;49:1242–7. 19233601 10.1016/j.jvs.2008.11.069

[56] Melikian R, O’Donnell TF Jr, Suarez L, et al. Risk factors associated with the venous leg ulcer that fails to heal after 1 year of treatment. J Vasc Surg Venous Lymphat Disord. 2019;7:98–105. 30558732 10.1016/j.jvsv.2018.07.014

[57] Yang X, Wu X, Peng Z, et al. Outcomes of endovenous laser ablation with additional iliac vein stenting of nonthrombotic lesions in patients presenting with active venous ulcers. J Vasc Surg Venous Lymphat Disord. 2021;9:1517–25. 33957281 10.1016/j.jvsv.2021.04.013

[58] Pihlaja T, Vanttila L, Ohtonen P, et al. Factors associated with delayed venous ulcer healing after endovenous intervention for superficial venous insufficiency. J Vasc Surg Venous Lymphat Disord. 2022;10:1238–44. 35961629 10.1016/j.jvsv.2022.07.008

[59] Aleksandrowicz H, Owczarczyk-Saczonek A, Placek W. Venous leg ulcers: advanced therapies and new technologies. Biomedicines. 2021;9:1569. 34829797 10.3390/biomedicines9111569 PMC8615583

[60] Elsharnoby AM, El-Barbary AH, Eldeeb A, et al. Resistant Chronic Venous Leg Ulcers: Effect of Adjuvant Systemic Hyperbaric Oxygen Therapy Versus Venous Intervention Alone. Int J Low Extrem Wounds. 2022;1–8. 10.1177/15347346221100891 35578536

[61] Cox C, Fry K, Sivakumaran Y, et al. Anti-IL17A, Ixekizumab, for treatment-resistant chronic venous leg ulcers: A phase II randomized, double-blind, placebo-controlled pilot trial. J Eur Acad Dermatol Venereol. 2023; 10.1111/jdv.19041. Epub ahead of print. PMID: 36914411. 10.1111/jdv.19041 36914411

[62] Pasek J, Cieślar G, Sieroń A. Combined therapy in the treatment of mixed etiology leg ulcer - case report. Ther Clin Risk Manag. 2018;14:1915–21. 30349270 10.2147/TCRM.S176321 PMC6183550

[63] Gillespie DL, Writing Group III of the Pacific Vascular Symposium 6. Kistner B, Glass C, et al. Venous ulcer diagnosis, treatment, and prevention of recurrences. J Vasc Surg. 2010;52:8S-14S. 10.1016/j.jvs.2010.05.068 20678885

[64] Margolis DJ, Berlin JA, Strom BL. Risk factors associated with the failure of a venous leg ulcer to heal. Arch Dermatol. 1999;135:920–6. 10456340 10.1001/archderm.135.8.920

[65] Falanga V. The chronic wound: impaired healing and solutions in the context of wound bed preparation. Blood Cells Mol Dis. 2004;32:88–94. 14757419 10.1016/j.bcmd.2003.09.020

[66] Mertz PM, Eaglstein WH. The effect of a semiocclusive dressing on the microbial population in superficial wounds. Arch Surg. 1984;119:287–9. 6365033 10.1001/archsurg.1984.01390150029007

[67] Dagher FJ, Alongi SV, Smith A. Bacterial studies of leg ulcers. Angiology. 1978;29:641–53. 360884 10.1177/000331977802900901

[68] Jull A, Slark J, Parsons J. Prescribed exercise with compression vs compression alone in treating patients with venous leg ulcers: a systematic review and meta-analysis. JAMA Dermatol. 2018;154:1304–11. 30285080 10.1001/jamadermatol.2018.3281 PMC6248128

[69] Marston W, Tang J, Kirsner RS, et al. Wound Healing Society 2015 update on guidelines for venous ulcers. Wound Repair Regen. 2016;24:136–44. 26663616 10.1111/wrr.12394

